# TM4SF1, a binding protein of DVL2 in hepatocellular carcinoma, positively regulates beta‐catenin/TCF signalling

**DOI:** 10.1111/jcmm.14787

**Published:** 2019-12-26

**Authors:** ChuanrRong Zhu, XiaoLing Luo, JinSheng Wu, YuTing Liu, Lei Liu, ShiJie Ma, Rui Xie, ShaoChuang Wang, Wu Ji

**Affiliations:** ^1^ Department of General Surgery Jinling Clinical Medical College Nanjing Medical University Nanjing China; ^2^ Department of Hepatobiliary & Pancreatic Surgery The Affiliated Huai’an No.1 People’s Hospital of Nanjing Medical University Huai’an China; ^3^ Department of Gastroenterology The Affiliated Huai’an No.1 People’s Hospital of Nanjing Medical University Huai’an China

**Keywords:** hepatocellular carcinoma, migration and metastasis, TM4SF1, Wnt/beta‐catenin cascade

## Abstract

The interaction between Axin and DVL2 is critical for the breaking down of the beta‐catenin destruction complex and the activation of the Wnt/beta‐catenin cascade. However, this biological process remains poorly understood. In the present study, TM4SF1 was identified as the interacting partner of DVL2 and positively regulated as Wnt/beta‐catenin signalling by strengthening the DVL2‐Axin interaction. The expression levels of TM4SF1 were elevated in hepatocellular carcinoma (HCC) and were induced by Kras signalling. The overexpression of TM4SF1 promoted the growth and motility of HCC cells, and up‐regulated the target genes (Axin2 and cyclin D1). The down‐regulation of TM4SF1 impaired the capability of HCC cells for growth, migration and metastasis. In addition, the down‐regulation of TM4SF1 promoted the ubiquitination of beta‐catenin. In summary, these results reveal the oncogenic functions of TM4SF1 in HCC progression and suggest that TM4SF1 might be a target for treatment.

## 1 INTRODUCTION

Multiple genetic alternations have been identified in the progression of hepatocellular carcinoma (HCC),^1^ such as the inactive mutations of tumour suppressors (SWI/SNF complex, Axin and P53),^2^ and the active mutations of oncogenes (Kras, beta‐catenin and so on).^3^ These genetic alternations lead to the dysregulation of cellular pathways (such as Kras signalling, Wnt/beta‐catenin signalling, P53 signalling, SWI/SNF signalling).^4^ Fully understanding the activation of these signals would benefit the treatment.

The nuclear accumulation of beta‐catenin has been frequently found in over half of patient‐derived HCC tissues.^5^ Beta‐catenin is at the central position of the Wnt/beta‐catenin signalling.^6^ The cellular destruction complex, which contains several scaffold proteins (APC and Axin) and kinases (CK1 and GSK3beta), tightly control the protein level of beta‐catenin.^7^ Upon stimulation of the receptor (LRP5/6 and Frizzled) with the Wnt ligands, Frizzled recruits DVL2.^8^ Then, Axin is recruited by DVL2 through the DIX domain, and the destruction complex is broken down, which leads to the accumulation of beta‐catenin in the cytoplasm. Thus, cytoplasmic beta‐catenin moves into the nucleus and promotes the transcription of downstream genes (Axin2, cyclin D1, c‐Myc and so on).^9^ The regulation for the interaction between Axin and DVL2 is critical for this cascade. Although several proteins have been identified to modulate this process, it remains not fully understood.^10^

As a tumour‐associated antigen, transmembrane 4 L6 family member 1 (TM4SF1) has been reported to be up‐regulated in most cancer types.^11^ Several studies have shown that TM4SF1 promotes the malignant phenotype of numerous cancer types, such as colon cancer,^12^ breast cancer,^13^ oesophageal cancer,^14^ pancreatic cancer.^15^ In the deep sequencing and comprehensive expression analysis, TM4SF1 was shown to be associated with human poorly differentiated HCC.^16^ In addition, the expression of TM4SF1 was regulated by microRNA‐520f.^17^ However, the functions and mechanism of TM4SF1 in promoting HCC remains not fully understood.

In the present study, the expression pattern, functions and mechanisms of TM4SF1 in HCC were evaluated.

## 2 MATERIALS AND METHODS

### 2.1 Cell culture

The HCC cells were obtained from the cell bank in the Chinese Academy of Science. These cells were cultured in Dulbecco's Modified Eagle's Medium (DMEM) with 10% foetal bovine serum (FBS) and antibiotics (penicillin and streptomycin) at a 37°C atmosphere with 5% CO_2_.

### 2.2 Clinical sample

The clinical samples were obtained from Huai'an First People's Hospital (Nanjing Medical University) with a written consent. These samples were stored at −80°C until use. The histopathology was evaluated by two pathologists.

### 2.3 Quantitative PCR (qPCR)

Trizol (Invitrogen, USA) was used to extract the total RNA from the tissues, according to manufacturer's instructions. The cDNA was produced using a Random Primers kit (Promega). The qPCR was performed using a 2 × SYBR Green mixture (Takara). The ribosomal protein Actin mRNA level acted as the internal control. The primer sequences for TM4SF1 were as follows: forward, 5’‐gcggctaatattttgcttta‐3’; reverse, 5’‐ccaatacagaagaaagcatc ‐3’.

### 2.4 Immunohistochemistry (IHC)

The sections were deparaffinized and rehydrated using xylene and ethanol, and 0.3% H_2_O_2_ solution was used to block the endogenous peroxidase activity. Then, the antigens were retrieved using sodium citrate solution (pH 6.0), and non‐specific binding was blocked using 5% BSA solution. Next, the sections were stained with TM4SF1 antibody and visualized with the secondary antibody (Envision, Gene Technology). Then, the slides were developed with DAB and counterstained with haematoxylin.

### 2.5 HCC mouse model

Alb‐Cre; LSL‐Kras^G12D^; P53^f/f^ mice (C57 background) were used as the HCC mouse model, which were housed with standard dark/light intervals (12‐h/12‐h). After four months, mice with HCC were killed, and the liver tissues were collected.

### 2.6 Cell transfection

Cells were plated in 35‐mm dish at a density of 5 × 10^5^/dish. Lipofectamine 2000 was used to deliver the plasmids to the HCC cells, according to the manufacturer's instructions. For the establishment of stable cell lines, the transfected cells were incubated with puromycin for one week, and the protein expression of exogenous TM4SF1 was examined using Western blot.

### 2.7 MTT assay

Briefly, 1000 cells per cells were seeded in a 96‐well plate and incubated with a total of 200 µL of medium. On the next day, these cells were incubated with an additional 20 µL of MTT solution for four hours. After the supernatant was removed, the OD 540 nm value was examined.

### 2.8 Cell migration assay

In the upper chamber, 1 × 10^5^ cells with 50 µL of medium (containing 0.1% FBS) were added. In the wells bellow, 150 µL of medium with 10% FBS was loaded. A membrane with 8‐µm pores was inserted between the upper and below chamber. After eight hours of incubation, the migrated cells were stained with eosin and photographed.

### 2.9 Soft agar

The bottom layer contained 0.5% agarose and 10% FBS in DMEM, and was used to coat the 12‐well plate. The upper layer of the 12‐well plates contained 0.35% agarose and 10% FBS in DMEM, and 2 × 10^3^ cells were suspended in the upper layer. The colonies were photographed and counted after 14 days of incubation. All experiments were performed for at least three times.

### 2.10 In vivo metastasis assay

The HCC cell line 7404 was labelled with luciferase and infected with the sh‐con lentivirus or sh TM4SF1 lentivirus. Tail vein injection was performed using the 1 × 10^6^ cells per nude mice. The metastasis was evaluated using the in vivo image system.

### 2.11 GST fusion protein pull‐down assay

The fusion protein GST‐TM4SF1 was expressed in *Escherichia coli* (*E coli*), captured by the sepharose 4B beads, and added to the cell lysates overnight. Then, the binding protein of GST or GST‐TM4SF1 was eluted with loading buffer and examined using Western blot.

### 2.12 Immunoprecipitation assay

The indicated plasmids were transfected into cells using Lipofectamine 6000. The transfected cells were harvested using the lysis buffer. After centrifugation, the supernatant was collected, and the primary antibody was added. After incubation overnight, the primary antibody was pulled down by protein A beads. Then, after four hours, the protein A beads were harvested and washed. Afterwards, the binding protein was eluted with the loading buffer and examined by Western blot.

### 2.13 Western blot

RIPA buffer was used to lyse the cells on ice for 20 minutes. The cell lysis was centrifuged at 10 000 *g* for 20 minutes at 4°C. Then, the supernatant was collected, and the concentration was measured using the Bradford assay. Afterwards, sodium dodecyl sulphate‐polyacrylamide gel electrophoresis (SDS‐PAGE) was performed. The polyvinylidene fluoride (PVDF) membrane was used for the protein transferred from the SDS‐PAGE for one hour, which was blocked with 5% milk (1 hour; room temperature) and incubated with the primary antibodies for at least eight hours. After washing for 2‐3 times, the membrane was incubated with the HRP‐coupled IgG at room temperature for one hour. After washing, the signals were detected using the ECL kit.

### 2.14 Reporter assay

Cells were seeded at a density of 2 × 10^4^ cells/well in a 24‐well plate. After 18 hours, cells reached 80% confluence. For each well, cells were transfected with 0.5 µg of TM4SF1 expression vector, 0.05 µg of Topflash and 0.01 µg of TK Renilla. The Fopflash was used as the control. After 24 hours, the reporter activity was measured using a dual reporter assay kit (Promega).

### 2.15 Ubiquitin assay

Cells were harvested using the lysis buffer after treatment with MG132 (10 µmol/L) for 10 hours. After centrifugation, the supernatant was collected, the beta‐catenin antibody was added, and the immunoprecipitation assay was performed. The immunoprecipitated protein was subjected to Western blot and examined using anti‐ubiquitin antibody.

## 3 RESULTS

### 3.1 HCC tissues had higher TM4SF1 expression

**Figure 1:**
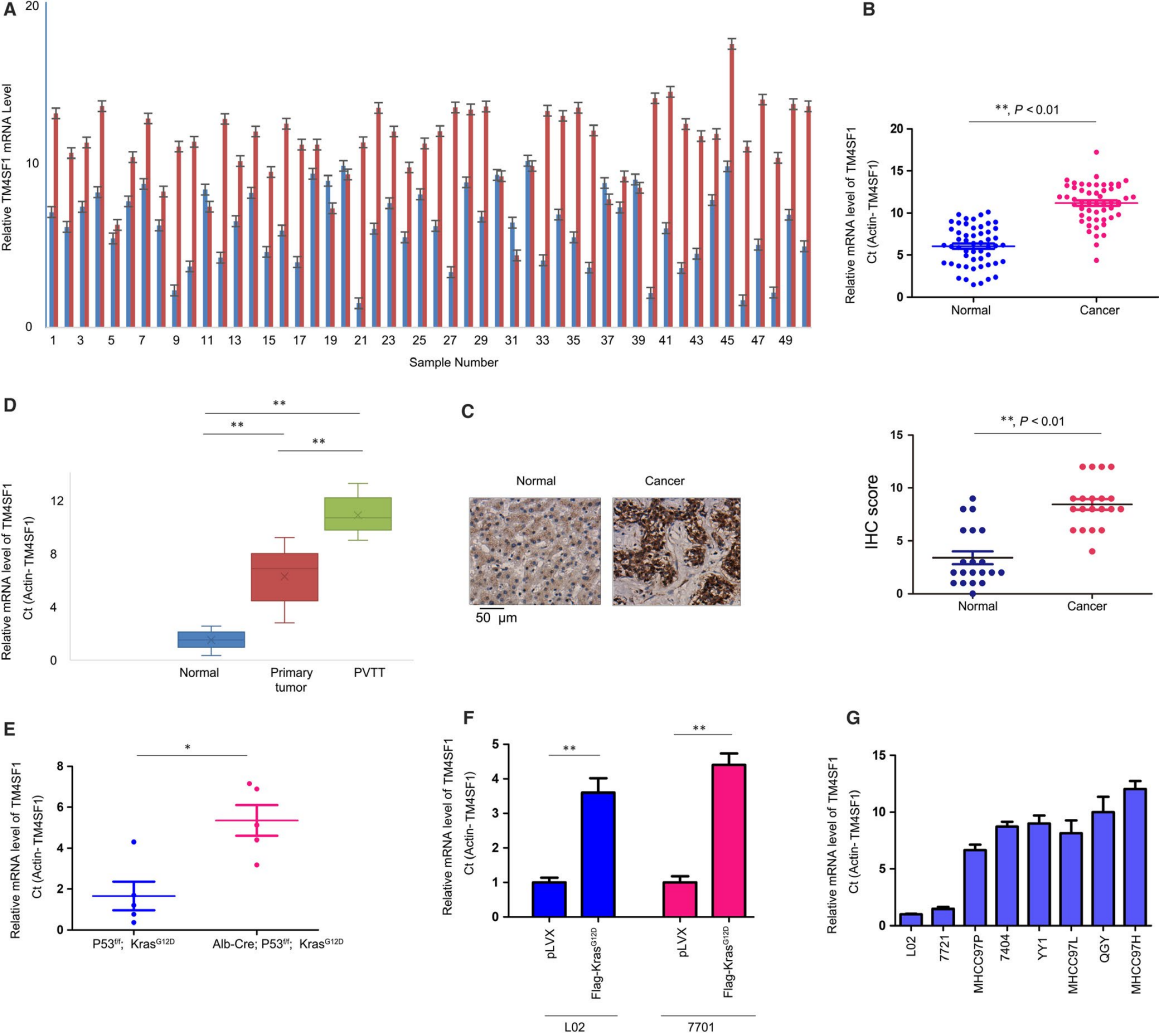
The mRNA and protein levels of TM4SF1 were elevated in HCC. A, The mRNA levels of TM4SF1 in the 50 HCC samples and paired non‐cancerous tissues were determined using qPCR. Actin was used as the internal control. Data were calculated as Ct (Actin‐TM4SF1). B, The statistical analysis of (A). C, Immunohistochemistry was performed to examine the protein level of TM4SF1 in HCC tissues. The statistical analysis was performed. D, The mRNA levels of TM4SF1 in the portal vein thrombus. The HCC samples and paired non‐cancerous tissues were determined by qPCR. E, The mRNA levels of TM4SF1 in the liver of Alb‐Cre; P53^f/f^; Kras^G12D^ mice and control mice were examined by qPCR. F, L02 and 7404 cells were transformed with Kras^G12D^, and the mRNA levels of TM4SF1 were examined by qPCR. G, The mRNA levels of TM4SF1 in the cell lines were determined by qPCR. *P < .05 and **P < .01; Scale bar = 50 µm

The RNA of 50 HCC tissues and paired non‐cancerous tissues were extracted using Trizol, and the transcripts of TM4SF1 were examined by qPCR. The upregulation of TM4SF1 was found in 80% of HCC tissues (Figure 1A,B). The observations from the IHC further confirmed the higher TM4SF1 protein level in these cancerous tissues (Figure 1C). Portal vein tumour thrombus (PVTT) has been recognized as a metastatic form of this malignancy. Hence, the transcripts of TM4SF1 in normal, primary HCC and PVTT tissues were evaluated. The expression of TM4SF1 was gradually elevated during the progression of this malignancy (Figure 1D).

The active mutation of Kras and inactive mutation of P53 are frequently found in HCC.^18^ The Alb‐Cre; LSL‐Kras^G12D^; P53^f/f^ mouse model has been widely used to study HCC. Hence, the expression of TM4SF1 in Alb‐Cre; LSL‐Kras^G12D^; P53^f/f^ mice and control mice was examined. Similarly, a higher TM4SF1 mRNA level was found in the HCC mouse model (Figure 1E). Next, it was determined whether Kras^G12D^ induced the expression of TM4SF1. L02 and 7701 cells were transfected with exogenous Kras^G12D^ plasmids (Flag‐Kras^G12D^). Indeed, the overexpression of Kras^G12D^ increased the mRNA level of TM4SF1 (Figure 1F). Finally, the TM4SF1 mRNA levels in HCC and normal cell lines (L02) were examined, and L02 cells were found to have lower TM4SF1 mRNA levels (Figure 1G). Collectively, these data demonstrate the upregulation of TM4SF1 in HCC.

### 3.2 The promoting effects of TM4SF1 on the growth and motility of HCC cells

**Figure 2:**
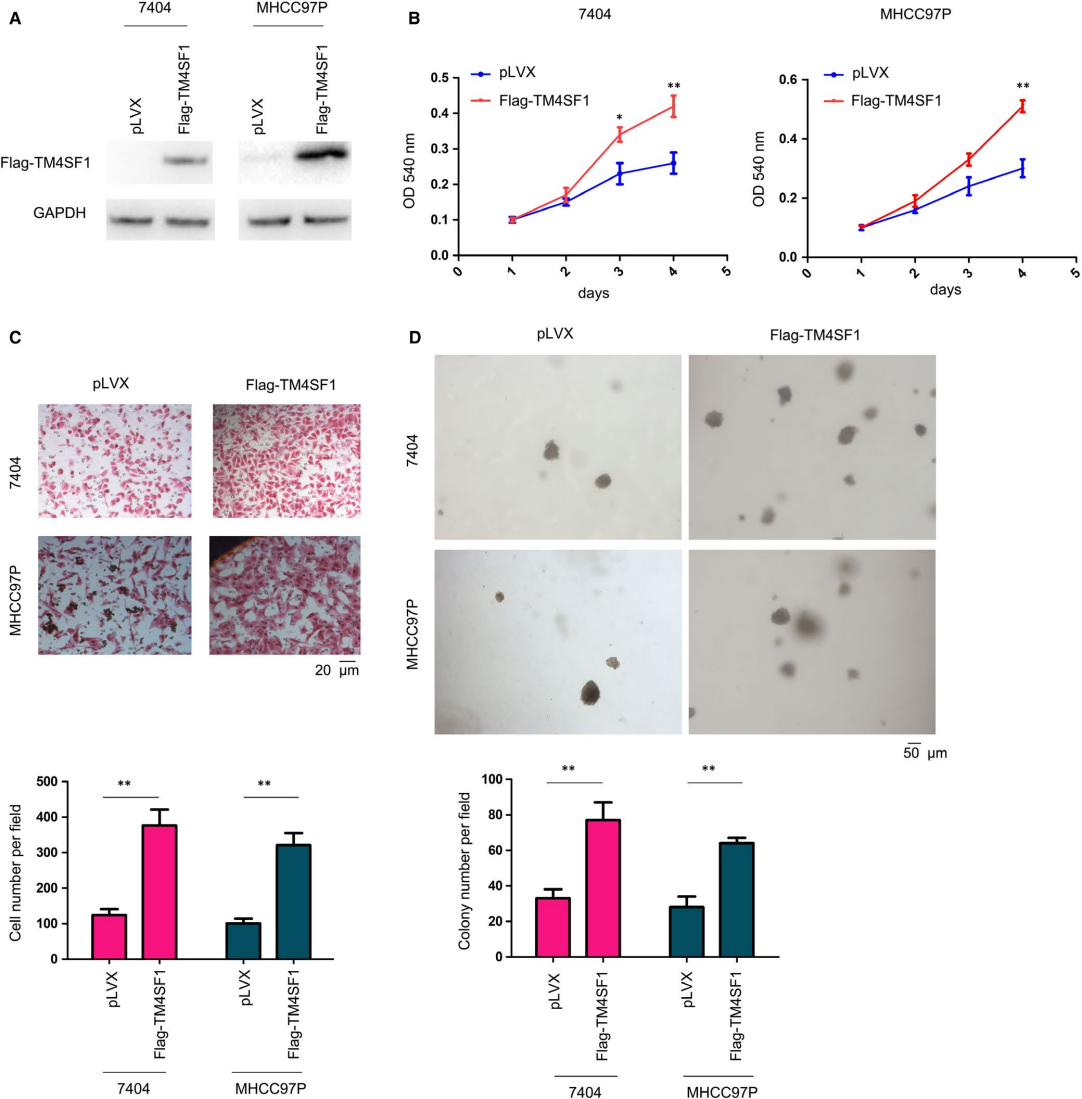
TM4SF1 promoted the growth, migration and colony formation of HCC cells. A, The overexpression of TM4SF1 in 7404 and MHCC97P cells. The expression of TM4SF1 was examined by Western blot. B, The MTT assay was performed to measure the growth of HCC cells. C, Cell migration capacity was evaluated using a Boyden chamber. Scale bar = 20 µm. D, An anchorage‐independent assay was performed. Scale bar = 50 µm. *P < .05 and **P < .01

**Figure 3:**
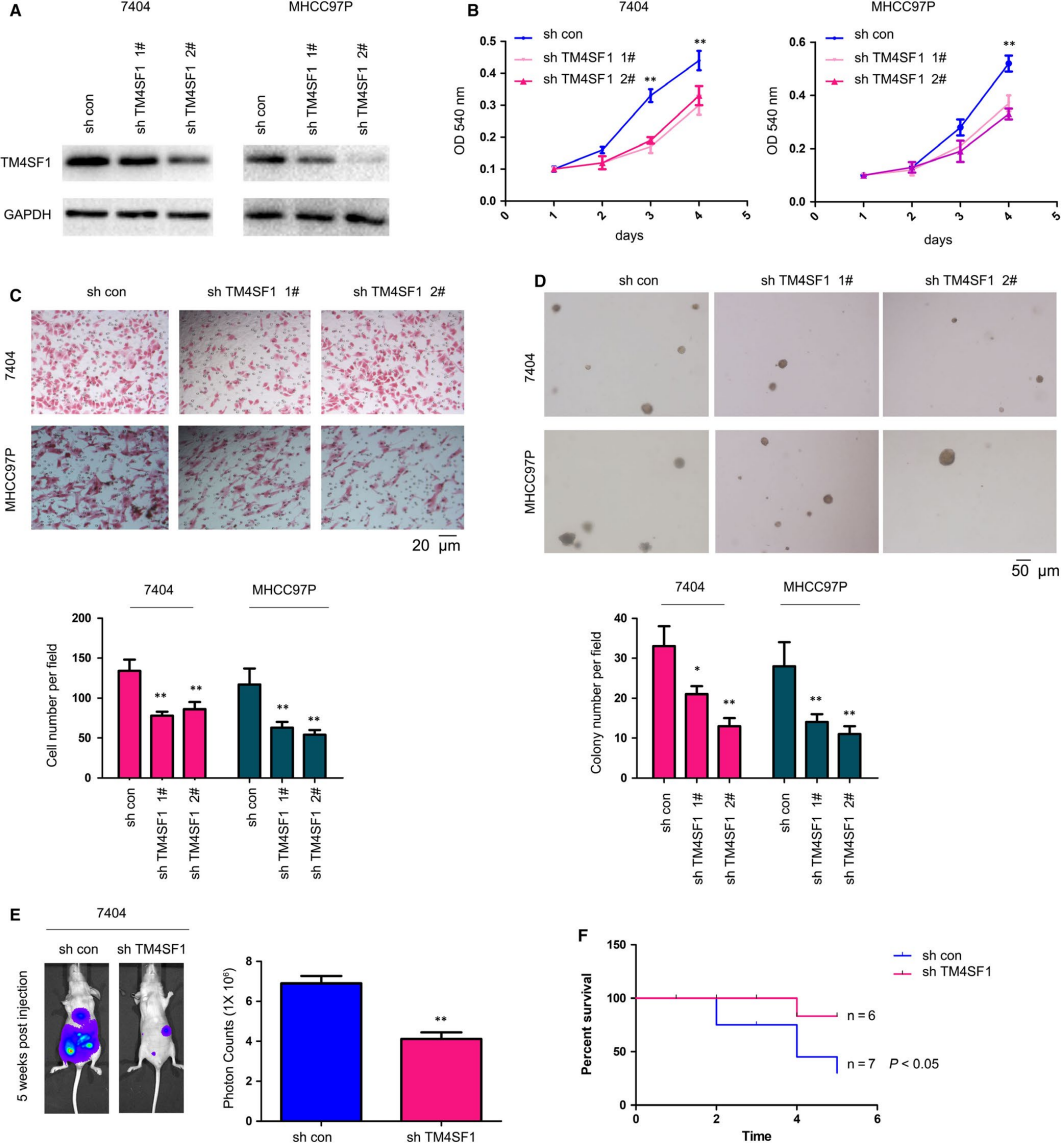
Knocking down the expression of TM4SF1 inhibited the growth, migration, metastasis and colony formation of HCC cells. A, The knockdown of the expression of TM4SF1 in 7404 and MHCC97P cells. The expression of TM4SF1 was examined by Western blot. B, The MTT assay was performed to measure the growth of HCC cells. C, Cell migration capacity was evaluated using a Boyden chamber. Scale bar = 20 µm. D, The anchorage‐independent assay was performed. Scale bar = 50 µm. E, The effects of TM4SF1 expression on the distant seeding of HCC cells were evaluated. Cells were injected into nude mice through the tail vein, and the metastatic foci were monitored using the in vivo system. F, The survival analysis of mice in (E). *P < .05 and **P < .01

Gain‐of‐function assay was performed by establishing 7404 and MHCC97P cells that were stably expressing Flag‐TM4SF1 (Figure 2A). The functions of TM4SF1 in the growth and motility of HCC cells were evaluated. Stably transfected 7404 and MHCC97P cells with TM4SF1 promoted cell growth and the migration of HCC cells (Figure 2B,C). Furthermore, TM4SF1 promoted the colony formation of HCC cells on the soft agar (Figure 2D). In order to further confirm the observations above, TM4SF1 was knocked down in 404 and MHCC97P cells (Figure 3A). The knockdown of the expression of TM4SF1 impaired the growth, migration and colony formation of HCC cells (Figure 3B‐D).

The regulation of cell migration by TM4SF1 prompted the investigators to determine whether the knockdown of TM4SF1 inhibited the metastasis of HCC cells. Indeed, the knockdown of TM4SF1 attenuated the distant seeding of 7404 cells (Figure 3E). Consistently, mice injected with 7404 cells, in which the expression of TM4SF1 was knocked down, exhibited the poorer survival (Figure 3F). These data suggest that TM4SF1 promoted the malignant behaviours of HCC cells.

### 3.3 The overexpression of TM4SF1 led to the accumulation of beta‐catenin in HCC cells

**Figure 4:**
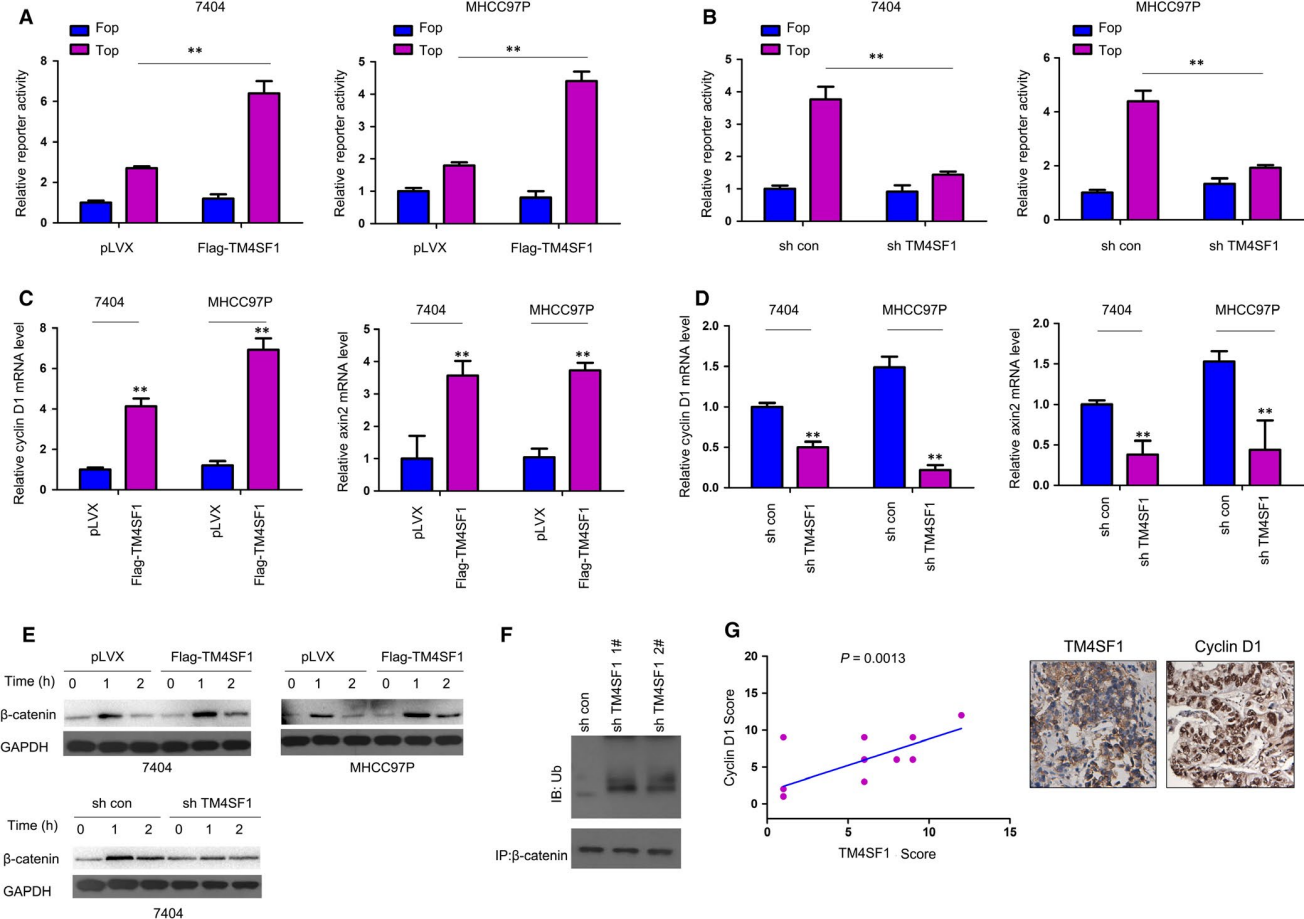
TM4SF1 activated the beta‐catenin/TCF signalling pathway in HCC. A‐B, The effects of TM4SF1 expression on the activity of the Topflash reporter. Fopflash reporter was used as a negative control. C‐D, QPCR was performed to evaluate the effects of TM4SF1 on the expression of cyclin D1 and Axin2. E, The effects of TM4SF1 expression on the accumulation of beta‐catenin were examined in 7404 and MHCC97P cells. Cells were treated with the Wnt3a conditioned medium, and the protein level of beta‐catenin was examined. F, The effects of TM4SF1 on the ubiquitination of beta‐catenin were examined. Cells were treated with MG132 for 10 h, and the cell lysates were immunoprecipitated with beta‐catenin antibody. After SDS‐PAGE, the ubiquitination was performed. G, The correlation between the expression of cyclin D1 and TM4SF1. The representative images were shown. **P < .01

In order to screen the pathways regulated by TM4SF1, a luciferase assay was performed. It was found that TM4SF1 promoted the activation of Topflash (an indicator of beta‐catenin transcriptional activity, Figure 4A). However, the knockdown of TM4SF1 decreased the Topflash reporter activity (Figure 4B). Consistently, the overexpression of TM4SF1 increased the mRNA levels of Axin2 and cyclin D1 (two downstream genes of beta‐catenin), while the knockdown TM4SF1 decreased the Axin2 and cyclin D1 mRNA level (Figure 4C,D). Furthermore, the overexpression of TM4SF1 potentiated the accumulation of beta‐catenin when cells were treated with wnt3a, while the knockdown TM4SF1 impaired the beta‐catenin accumulation (Figure 4E). Moreover, the silencing TM4SF1 promoted the ubiquitination of beta‐catenin (Figure 4F), suggesting that TM4SF1 enhanced the stability of beta‐catenin. In addition, TM4SF1 expression positively correlated with that of cyclin D1 in HCC tissues (Figure 4G).

### 3.4 TM4SF1 strengthened the Axin‐DVL2 interaction

**Figure 5:**
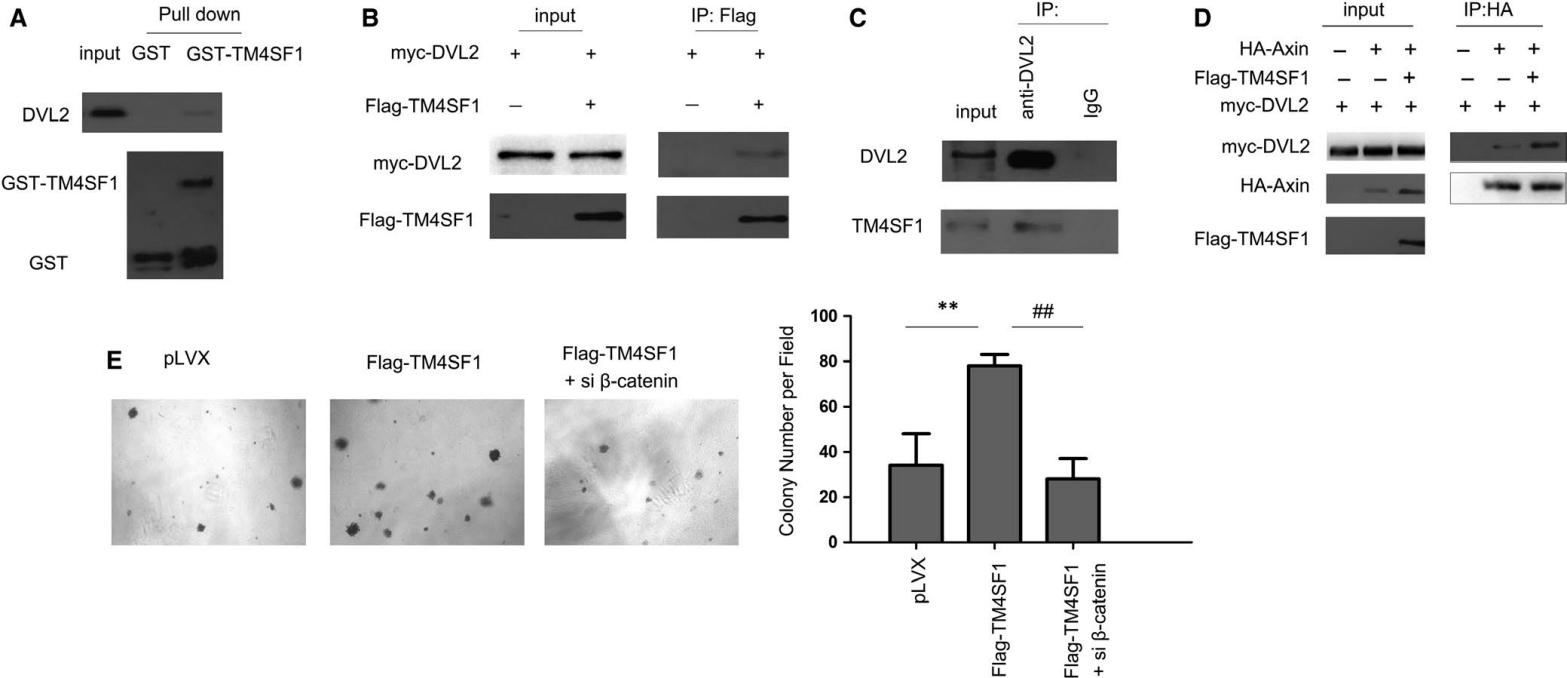
TM4SF1 interacted with DVL2 in HCC cells. A, GST pull‐down assay was performed to examine the interaction between DVL2 and TM4SF1. B, The immunoprecipitation assay was performed to examine the interaction between the exogenously expressed DVL2 and TM4SF1. The 7404 cells were transfected with myc‐DVL2 and Flag‐TM4SF1 plasmids. After 48 h, the immunoprecipitation assay was performed. C, The immunoprecipitation assay was performed to examine the interaction between the endogenously expressed DVL2 and TM4SF1. D, The immunoprecipitation assay was performed to examine the effects of TM4SF1 expression on the interaction between DVL2 and Axin. E, The knockdown of beta‐catenin rescued the colony formation induced by TM4SF1 in 7404 cells. *P < .05, **P < .01 and ##*P* < .01

In order to understand the details on how TM4SF1 regulated the stability of beta‐catenin, the interactions among the upstream components were examined. First, the fusion protein GST‐TM4SF1 was found to pull down the DVL2 protein (Figure 5A). The interaction between DVL2 and TM4SF1 was further demonstrated by the immunoprecipitation assay (Figure 5B,C). Furthermore, TM4SF1 strengthened the Axin‐DVL2 interaction (Figure 5D). Next, it was determined whether Wnt/beta‐catenin signalling mediated the biological function of TM4SF1. Beta‐catenin was knocked down to rescue the functions of TM4SF1. The knockdown of beta‐catenin impaired the anchorage‐independent growth of HCC cells driven by TM4SF1 (Figure 5E). In summary, these observations demonstrate that TM4SF1 strengthened the Axin‐DVL2 interaction, thereby stabilizing the beta‐catenin signal.

## 4 DISCUSSION

The accumulation of nuclear beta‐catenin can be found in more than a half of clinical HCC samples,^19^ which worsens the outcome of HCC patients. Upon the binding of Wnt ligands, the interaction of DVL2 and Axin relays signals from the membrane to the cytoplasm, which is critical for the disassociation of the beta‐catenin destruction complex. However, the details on DVL2‐Axin interaction regulation are not fully elucidated. In the present study, it was found that TM4SF1 strengthened the DVL2‐Axin interaction by directly binding to DVL2. TM4SF1 expression level was elevated in HCC and exaggerated the malignant behaviours of HCC cells, indicating that targeting TM4SF1 might benefit these patients.

The induction of TM4SF1 by Kras^G12D^ was demonstrated in the present study, which was supported by two observations: (a) the mRNA level of TM4SF1 was elevated in the HCC mouse model driven by P53 loss and Kras^G12D^; (b) Kras^G12D^ up‐regulated the mRNA level of TM4SF1 in L02 and 7404 cells. Kras^G12D^ mutation was prevalent in HCC. The present study established a link between Kras active mutation and beta‐catenin/TCF signalling, and indicated that targeting the TM4SF1‐mediated activation of the beta‐catenin/TCF cascade might be helpful for the therapy of Kras^G12D^‐driven cancer.

Another interesting finding was that the Axin‐DVL2 interaction was strengthened by TM4SF1. It has been reported that the DIX domains in both Axin and DVL2 mediated their interaction.^20^ Several proteins have been identified as modulators for the interaction between Axin and DVL2.^21^ The present study demonstrated that TM4SF1, a downstream effector of Kras, strengthened the Axin‐DVL2 interaction.

In summary, the present study illustrated the expression, functions and mechanism of TM4SF1 in HCC, and indicated that TM4SF1 might be a potential target.

## CONFLICT OF INTERESTS

The authors declare no conflict of interest with respect to this research.

## AUTHORS’ CONTRIBUTIONS

Ji Wu and Shaochuang Wang designed this study. Chuanrong Zhu, Xiaoling Luo, Jingsheng Wu, Yuting Liu, Liu Lei, Shijie Ma, and Xie Rui performed the experiments.

## ETHICS APPROVAL AND CONSENT TO PARTICIPATE

All experimental protocols were approved by the Institutional Committee of Nanjing Medical University. An informed consent was obtained from all subjects. The study was reviewed and approved by the China national institutional animal care and use committee.

## CONSENT FOR PUBLICATION

All authors agreed on the manuscript.

## Data Availability

The data that support the findings of the present study are available from the corresponding author upon reasonable request.
